# Impact of HIV-1 infection on mortality among new diagnosed cases in a hospital in Bucharest

**DOI:** 10.1186/1471-2334-14-S4-P12

**Published:** 2014-05-29

**Authors:** Simona Erscoiu, Ionuț Cristian Popa, Olivia Burcoş, Raia Loghin, Ana Maria Mehedinți, Emanoil Ceauşu

**Affiliations:** 1 Carol Davila University of Medicine and Pharmacy, Bucharest, Romania; 2 Clinical Hospital of Infectious and Tropical Diseases “Dr. Victor Babeş”, Bucharest, Romania

## 

According to new published data, HIV/AIDS infection currently knows an increase of 8% for the countries of Eastern Europe and Central Asia. This happened in our country too, where a new way to get started HIV appeared: intravenous drugs used of so-called soft, legal drugs (ethnobotanicals) near the classic heroin. Objectives: To study the impact of HIV infection and the trend in mortality in a cohort of 497 newly diagnosed patients enrolled between 01.01.2011 and 31.12.2013 in the Clinical Hospital of Infectious and Tropical Diseases “Dr. Victor Babeş”, Department Casa Andreea, Bucharest. These new cases were intravenous drug users (IVDUs) but also late and very late presenters sexually infected and nonIVDUs.

We performed a retrospective study of patients newly diagnosed with HIV infection that died during the 36 analyzed months. The research was based on clinical records and autopsy reports. Patients lost from follow up or died at home were excluded.

Our cohort enrolled 497 patients: 249 IVDUs and 248 nonIVDUs. Men were in greater proportion, dominated the IVDU group. The average patient age was 30.8 years old, similar age for both genders. Sixty-eight (13.65%) patients died. 27 IVDU and 41 nonIVDU (total deaths were 106 - 20.1% those diagnosed before 2011 but who died in the analyzed period were excluded). Most deaths were recorded in IVDU in 2012, and in nonIVDU in 2011. Mean CD4 cell count was 126.62 lymphocytes/cmm in IVDU and 75.75/cmm in nonIVDU. IVDUs were classified in approximately equal proportions in C3/B3 class and the other category in class C3. HCV co infection was present in all IVDU and only in 7.31% of nonIVDUs. IVDUs causes of death in descending order were MSSA sepsis and tricuspid endocarditis (in 2013 began appearing MRSA) followed by pulmonary and disseminated tuberculosis (TB), HCV decompensated cirrhosis and drugs overdoses. In nonIVDUs, opportunistic infections were causes of death (toxoplasmosis, PCP, neglected TB treatment, lymphoma). Death occurred at a mean of 14.5 weeks (limit 3 days-104 weeks) for nonIVDU and 17.7 weeks for the IVDU weeks (range: 1 day- 93 weeks) for IVDUs.

